# Biomarkers of Lead Exposure and DNA Methylation within Retrotransposons

**DOI:** 10.1289/ehp.0901429

**Published:** 2010-01-11

**Authors:** Robert O. Wright, Joel Schwartz, Rosalind J. Wright, Valentina Bollati, Letizia Tarantini, Sung Kyun Park, Howard Hu, David Sparrow, Pantel Vokonas, Andrea Baccarelli

**Affiliations:** 1 Department of Environmental Health, Harvard School of Public Health, Boston, Massachusetts, USA; 2 Department of Pediatrics, Children’s Hospital Boston, Harvard Medical School, Boston, Massachusetts, USA; 3 The Channing Laboratory, Brigham and Women’s Hospital, Harvard Medical School, Boston, Massachusetts, USA; 4 Laboratory of Environmental Epigenetics, Department of Occupational and Environmental Health, IRCCS Maggiore Policlinico Hospital, Mangiagalli e Regina Elena Foundation and Università degli Studi di Milano, Milan, Italy; 5 Department of Environmental Health Sciences, University of Michigan School of Public Health, Ann Arbor, Michigan, USA; 6 VA Normative Aging Study, VA Boston Healthcare System and Department of Medicine, Boston University School of Medicine, Boston, Massachusetts, USA

**Keywords:** aging, DNA methylation, epigenetics, lead, metals

## Abstract

**Background:**

DNA methylation is an epigenetic mark that regulates gene expression. Changes in DNA methylation within white blood cells may result from cumulative exposure to environmental metals such as lead. Bone lead, a marker of cumulative exposure, may therefore better predict DNA methylation than does blood lead.

**Objective:**

In this study we compared associations between lead biomarkers and DNA methylation.

**Methods:**

We measured global methylation in participants of the Normative Aging Study (all men) who had archived DNA samples. We measured patella and tibia lead levels by K-X-Ray fluorescence and blood lead by atomic absorption spectrophotometry. DNA samples from blood were used to determine global methylation averages within CpG islands of long interspersed nuclear elements-1 (LINE-1) and Alu retrotransposons. A mixed-effects model using repeated measures of Alu or LINE-1 as the dependent variable and blood/bone lead (tibia or patella in separate models) as the primary exposure marker was fit to the data.

**Results:**

Overall mean global methylation (± SD) was 26.3 ± 1.0 as measured by Alu and 76.8 ± 1.9 as measured by LINE-1. In the mixed-effects model, patella lead levels were inversely associated with LINE-1 (β = −0.25; *p* < 0.01) but not Alu (β = −0.03; *p* = 0.4). Tibia lead and blood lead did not predict global methylation for either Alu or LINE-1.

**Conclusion:**

Patella lead levels predicted reduced global DNA methylation within LINE-1 elements. The association between lead exposure and LINE-1 DNA methylation may have implications for the mechanisms of action of lead on health outcomes, and also suggests that changes in DNA methylation may represent a biomarker of past lead exposure.

Epigenetics can be defined broadly as the study of the temporal and spatial regulation of gene expression. Changes in epigenetic marks can be as profound as DNA sequence mutations, but unlike DNA mutations, epigenetic marks are reversible and responsive to the environment (Baccarelli and Bollati 2009). DNA methylation is the best studied of the epigenetic processes that regulate gene expression. DNA methylation typically occurs in cytosines within CpG repeat sequences found in gene promoter regions. Areas enriched in CpG repeats are frequently referred to as “CpG islands.” Methylation of DNA within promoter regions regulates local gene transcription. In general, increased DNA methylation is inversely associated with gene expression. Much epigenetic research has been focused on the heritability of phenotypic traits not attributable to alterations in DNA sequence, but such epigenetic modifications would have to occur in germ cells to be truly heritable. Changes in epigenetic marks in somatic cells are also of great interest, as these changes can also be induced by environmental exposures and may play a role in the toxicity of environmental agents, even if they are not heritable.

Previous research in both animals and humans has noted that DNA methylation can be altered by exposure to environmental metals (Reichard et al. 2007; Sciandrello et al. 2004; Takiguchi et al. 2003). Oxidative stress may be a unifying process to explain these findings across different metals. Metals are known to increase production of reactive oxygen species in a catalytic fashion via redox cycling (Fowler et al. 2004; Valko et al. 2005). Oxidative DNA damage can interfere with the ability of methyltransferases to interact with DNA (Turk et al. 1995; Valinluck et al. 2004), thus resulting in a generalized hypomethylation of cytosine residues at CpG sites (Turk et al. 1995). Long-term exposure to oxidative stress has been shown to result in oxidative damage of methylated cytosine residues and depletion in the level of 5-methylcytosine in repeated elements (Pogribny et al. 2007; Valinluck et al. 2004). Lead is among the most prevalent of toxic environmental metals, has substantial oxidative properties, and could also alter epigenetic marks after long-term exposure.

DNA methylation can be measured both in specific genes and using global DNA methylation markers such as repetitive DNA sequences (Bollati et al. 2007; Kazazian and Moran 1998; Pilsner et al. 2007; Roman-Gomez 2005). Measures of global methylation typically involve methylation within repetitive DNA sequence. The two primary types of repetitive elements studied are Alu and long interspersed nucleotide elements (LINE-1) (Bollati et al. 2007; Issa 2002; Pilsner et al. 2007). There are approximately 1.4 million Alu repetitive elements in the human genome and half a million LINE-1 elements. LINE-1 and Alu are sometimes referred to as “junk” DNA. They are remnants of viral DNA (transposons) or viral RNA (retrotransposons) that were incorporated into the human genome over evolutionary history (Carnell and Goodman 2003; Wallace et al. 2008a, 2008b). If transcribed, such elements can reinsert into DNA, potentially moving across the genome. These elements are rich in CpG repeats and are typically highly methylated in order to suppress their expression (Schulz et al. 2006). Because such elements represent approximately half of all DNA sequences, changes in methylation in LINE-1 and Alu are believed to be indicative of global or genomic methylation (Bestor and Tycko 1996; Klose and Bird 2006).

In this study, we analyzed global DNA methylation markers (Alu and LINE-1) in a cohort of elderly men who have been well characterized for their environmental cumulative exposure to lead. Because of the stable, yet modifiable nature of DNA methylation, we hypothesized that bone lead, a marker of cumulative exposure with a half-life measured in years, would be associated with changes in global DNA methylation and that this association would be more evident than an association with a biomarker of concurrent/short-term exposure such as blood lead.

## Methods

This study was approved by the institutional review boards of Harvard School of Public Health, Brigham and Women’s Hospital, and the Boston Veteran’s Administration Hospital, and all participants gave written informed consent. This study was conducted on a subsample of the Normative Aging Study (NAS), a multidisciplinary longitudinal study of aging established by the Veterans Administration in 1963 (Bell et al. 1966). Briefly, 2,280 men were enrolled in the NAS. Participants received their first medical examination between 1963 and 1969. Subsequently, subjects have reported for medical examinations and standard blood and urine tests every 3–5 years. During those visits, NAS participants filled out questionnaires on smoking history, education level, food intake, and other risk factors that may influence health. Beginning in 1991, those who gave their informed consent presented to the Ambulatory Clinical Research Center of Brigham and Women’s Hospital for a K-X-ray fluorescence (KXRF) measurement of lead content in the tibia and patella. Study subjects were thus measured for bone lead between 1991 and 1999.

### Bone lead levels measured by KXRF

Bone lead measurements were taken at two sites, the mid-tibial shaft and the patella, with an ABIOMED KXRF instrument (ABIOMED, Inc., Danvers, MA, USA, and the Harvard Metals Epidemiology Research Group) (Hu et al. 1990). The tibia and patella have been targeted for bone lead research because these two bones consist mainly of pure cortical and trabecular bone, respectively. A 30-min measurement was taken at the midshaft of the left tibia and at the left patella after each region had been washed with a 50% solution of isopropyl alcohol. The tibial midshaft was taken as the point equidistant between the tibial plateau and the medial malleolus. The KXRF beam collimator was sited perpendicular to the flat bony surface of the tibia and at the patella.

As a quality-control measure, once a week a 15-ppm phantom was positioned and measured 20 consecutive times overnight as a first-order calibration check. Analysis of means and SDs was performed to disclose any significant shift in accuracy or precision. Once a month, the entire set of calibration phantoms (0, 5, 10, 15, 20, 30, 40, 50, and 100 ppm; true values checked by inductively coupled plasma-mass spectrometry) was measured, and a calibration curve was calculated as a final check on calibration.

### Blood lead levels measured by graphite atomic absorption spectrometry

Fresh blood for lead measurement was taken in a special lead-free tube containing EDTA and sent to ESA Laboratories, Inc. (Chelmsford, MA, USA). Blood samples were analyzed by a Zeeman background-corrected flameless atomic absorption spectrophotometer (graphite furnace). The instrument was calibrated before use with National Institute of Standard and Technology materials. Ten percent of the samples were run in duplicate. In tests on reference samples from the Centers for Disease Control and Prevention, precision (% relative SD) ranged from 8% for concentrations < 30 μg/dL to 1% for higher concentrations. Blood lead levels are measured at each triennial NAS study visit. For purposes of this study, we used the blood lead measurement most proximal in time to the bone lead measurement.

### DNA methylation

#### Bisulfite treatment

DNA was extracted from stored frozen buffy coat of 7 mL whole blood, using the QiAmp DNA blood kits (QIAGEN, Valencia, CA, USA); 500 ng DNA (50 ng/μL) was treated using the EZ DNA Methylation-Gold Kit (Zymo Research, Orange, CA, USA) according to the manufacturer’s protocol. Final elution was performed with 30 μL M-Elution buffer.

#### Polymerase chain reaction and pyrosequencing

DNA methylation was quantitated using bisulfite-polymerase chain reaction (PCR) and pyrosequencing, using primers and conditions described previously (Baccarelli et al. 2009; Colella et al. 2003; Tarantini et al. 2009; Yang et al. 2004). We used LINE-1 and Alu element PCR for pyrosequencing-based methylation analysis following previously published methods (Bollati et al. 2007; Tost et al. 2003), with the following modifications: A 50-μL PCR was carried out in 25 μL GoTaq Green Master mix (Promega, Madison, WI, USA), 1 pmol biotinylated forward primer, 1 pmol reverse primer, 50 ng bisulfite-treated genomic DNA, and water. A biotin-labeled primer was used to purify the final PCR product using Sepharose beads. The PCR product was bound to Streptavidin Sepharose HP (Amersham-Biosciences, Uppsala, Sweden), and the Sepharose beads containing the immobilized PCR product were purified, washed, denatured using a 0.2-M NaOH solution, and washed again using the Pyrosequencing Vacuum Prep Tool (Pyrosequencing Inc., Westborough, MA), according to the manufacturer’s recommendations. Pyrosequencing primer (0.3 μM) was then annealed to the purified single-stranded PCR product, and pyrosequencing was performed using the Pyromark MD System (Pyrosequencing Inc.). The degree of methylation was expressed as the percentage of 5-methylated cytosines (%5mC) divided by the sum of methylated and unmethylated cytosines. The assays, which allow for the amplification of a representative pool of repetitive elements, quantitatively assess the proportion of methylated sites in Alu and LINE-1 repetitive elements dispersed throughout the genome. Measures of Alu and LINE-1 methylation are highly correlated with 5-methylcytosine content measured through high-performance liquid chromatography and have been widely used as a surrogate of global methylation (Weisenberger et al. 2005; Yang et al. 2004). We used non-CpG cytosine residues as built-in controls to verify bisulfite conversion. Each sample was tested in two replicates, and their average was used in the statistical analysis.

### Statistical analysis

We excluded tibia and patella bone lead measurements with estimated uncertainties > 10 μg/g bone and > 15 μg/g bone, respectively, because these measurements usually reflect excessive patient movement during the measurement (Hu et al. 1998). Such procedures are standard in analysis of bone lead data (Aro et al. 1994). We used the generalized extreme studentized deviation many-outlier method (Rosner 1983) to remove extreme outliers among the continuous independent variables with a normal distribution, as performed in previous analyses (Carey et al. 1997; Hu et al. 1996; Rexrode et al. 1998; Whitsel et al. 2001). This procedure removed 10 subjects with extreme tibia lead levels and 8 subjects with extreme patella lead levels. We also report results of analyses in which those subjects with extreme bone lead measurements were included.

### Statistical analysis of predictors of DNA methylation levels

We fit a mixed-effects model to account for repeatedly measured methylation levels. Analyses were performed to evaluate the association between lead biomarkers and DNA methylation levels. The potential confounding factors included were age, body mass index (BMI), percentage of lymphocytes among white blood cells (WBCs), education, and pack-years of cigarettes. Blood lead levels were used as the index of lead exposure in a separate model. Adjustment for confounders was conducted in stages to identify which confounders had a strong influence on results. A random slope for the time elapsed from the first visit for DNA methylation was initially considered to account for subject–specific variability of methylation over time, but a random-intercept-only model was preferred based on the likelihood ratio test comparing the two models. We also examined whether the association between bone lead and global DNA methylation levels changed over time. We included an interaction term between the time elapsed from the first visit and each bone lead marker, but we did not see any statistically significant interaction. Therefore, we eliminated this interaction term from the final model. The following equation describes the structure of our fitted models:


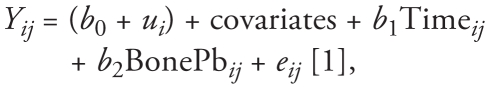


where *Y_ij_* is the methylation levels (Alu or LINE-1) in subject *i* at time *j*, *b*_0_ is the overall intercept, *u_i_* is the separate random intercept for subject *i*, *b*_1_ is the slope representing the overall effect of time, and *b*_2_ is the slope representing the overall effect of the bone lead marker. All mixed-effects models were conducted using the PROC MIXED procedure in SAS, version 9.1 (SAS Institute Inc., Cary, NC, USA).

To determine whether the associations between DNA methylation levels and the different lead biomarker were nonlinear, we also modeled the lead biomarker as a penalized spline in a generalized additive models using R software (R Foundation for Statistical Computing; http://www.r-project.org).

## Results

Between 1 March 1999 and 27 June 2007, 1,047 blood samples from 704 subjects were successfully analyzed for DNA methylation in both Alu and LINE-1. Of these 704 subjects, 168 did not have bone lead measurements, leaving 536 subjects available for analysis. Fourteen subjects with uncertainty > 10 g/g lead in bone for tibia or 15 g/g for patella were excluded, reducing this number to 522 subjects available for the analysis. Finally, 5 subjects with missing values in WBC differentials were also excluded. Therefore, in the end, 517 subjects with a total of 787 observations were available for analysis.

Table 1 lists the demographic characteristics of the population at baseline (i.e., at the first measurement of DNA methylation) and a comparison with those subjects who had bone lead data but did not supply DNA for methylation analysis. In general, subjects with and without methylation data were very similar with respect to the covariates. Table 2 presents the unadjusted relationships between lead biomarkers and global DNA methylation, as well as the relationship among the covariates and biomarkers of global DNA methylation at baseline. Consistent with our previous report (Bollati et al. 2009), DNA methylation values for both Alu and LINE-1 decreased with increasing age (Table 3). Trends are similar for increasing levels of patella lead, but no relationship is evident for either tibia or blood lead. A trend toward increased methylation at LINE-1 CpG islands was seen for both pack-years of cigarette smoking and increasing years of education, but not for Alu (Table 2).

**Figure 1:**
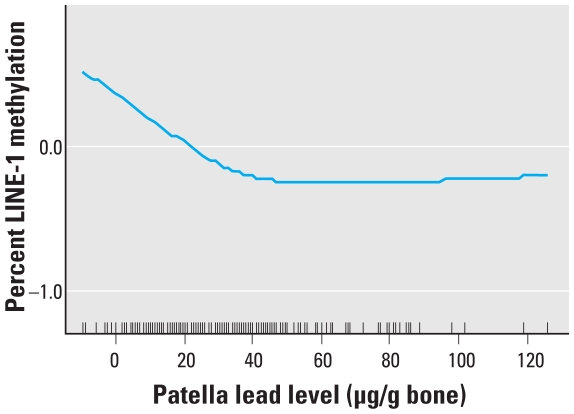
Penalized spline showing nonlinear relationship between patella lead level and LINE-1 DNA methylation. The plot is adjusted for age, BMI, education, WBC differential, and pack-years of smoking.

In our mixed-effects models adjusting for smoking, age, BMI, education, and the percentage of lymphocytes in the WBC differential, we found that, again, patella lead levels were associated with decreasing levels of global DNA methylation in LINE-1 retrotransposons but not with methylation at Alu elements (Table 3). We found no significant association between either global DNA methylation marker and blood or tibia lead levels. At baseline, LINE-1 methylation was inversely associated with age (Table 2). Finally, we generated a smoothed plot of the relationship between patella lead levels and LINE-1 element methylation (Figure 1). This plot suggests that the relationship may be nonlinear, with the primary decrease occurring between patella lead levels between 0 and approximately 40 μg/g bone and a leveling out of the effect at levels higher than this.

## Discussion

### Cumulative exposure to lead and DNA methylation

In the present study we found an association between patella lead levels (a biomarker of cumulative lead exposure) with LINE-1 repetitive element methylation. Blood lead levels (a biomarker of recent exposure) were not associated with DNA methylation. Our group (Pilsner et al. 2009) recently reported an association between global DNA methylation in umbilical cord WBC DNA and maternal patella lead levels. Here we expand that work by studying an aging population, as opposed to newborns and women of childbearing age. Our results suggest that cumulative past exposure to lead among elderly individuals is associated with decreased LINE-1 DNA methylation, even after adjusting for age. This association is therefore likely due to the effects of distant past exposure, rather than more contemporaneous lead exposure. Our study population is composed of elderly men, the majority of whom were retired at the time of the study, limiting any impact of recent or subacute occupational lead exposure not captured by blood or bone lead.

Bone lead is a unique biomarker in that past exposures can be accurately reconstructed and their impact on health directly measured. Because of bone lead’s half life of ≥ 10 years, ultimately > 90% of body lead is found in bone (Leggett 1993). Few chemicals have such a well-established cumulative exposure biomarker. This may mean that bone lead is uniquely suited as a paradigm biomarker in which to test whether DNA methylation patterns in WBCs correlate with lifetime environmental exposures. However, because bone is not a uniform tissue, we measured lead in two types of bone: trabecular and cortical. Tibia represents cortical bone, and its matrix turnover is slower than trabecular bone, which is represented by patella (Hu et al. 1998). Previous research shows that tibia lead is biologically more inert than patella lead but is a better dosimeter of cumulative exposure (Hu et al. 2007; Kosnett et al. 2007; Todd and Chettle 1994). This is reflected in the longer half-life of cortical lead levels (approximately 12–15 years) relative to trabecular lead levels (approximately 8–10 years). In general, the higher turnover rate of patella bone lead into blood means that lead within trabecular bone is more biologically active (Hu et al. 2007). This may explain why this particular biomarker was statistically associated with reduced methylation of LINE-1. Under the assumption that DNA-methylation changes in lymphocytes are most influenced by direct chronic exposure to circulating lead in blood, patella lead would be more likely to be associated with DNA methylation changes than tibia lead. Considering the differences in the kinetics of tibia and patella lead, the DNA methylation findings seen in this study suggest that redistribution of lead from bone over time may be part of the mechanism by which patella lead is associated with reduced DNA methylation in LINE-1. If the association were purely due to cumulative lead exposure, one would expect tibia lead to be more strongly associated with DNA methylation than patella lead.

### Global methylation markers

Changes in methylation patterns among markers of global methylation, such as LINE-1 and Alu, had been thought to represent a more passive response to environmental factors. Changes in methylation in these sequences could reflect the general impact of environmental factors on DNA methylation, as they were not believed to be functional and methylation would likely be less regulated compared with regions containing genes (Axume et al. 2007; Weisenberger et al. 2005). Such transposable elements comprise > 50% of DNA sequence and were initially believed to be nonfunctional “junk” DNA. More recent work has demonstrated that they play a critical role in human development, tissue differentiation, and gene expression, as these elements can be transcribed and translated into functional proteins (Belancio et al. 2009; Han and Boeke 2005). LINE-1 elements comprise about 20% of the human genome, are approximately 6,000 base pairs long, and consist of two open reading frames, one of which codes for an enzyme with both endonuclease and reverse transcriptase properties (Garcia-Perez et al. 2007; Wallace et al. 2008a, 2008b). Using this enzyme, LINE-1 elements can replicate and insert themselves into different genomic regions. Decreased methylation of LINE-1 elements would be expected to lead to the transcriptional activation of those repetitive sequences that contain complete promoters (Wilson et al. 2007). The majority of LINE-1 elements cannot be active because they have been either truncated or mutated over our evolutionary history. However, the human genome averages 500,000 LINE-1 copies, of which appproximately 3,000 LINE-1 sequences are complete (Lander et al. 2001) and 200 are potentially transcribed (Brouha et al. 2003). This is still a large number of biologically active retrotransposons. If demethylation of these LINE-1 elements activates their transcription and they are inserted near or within genes, they can alter gene expression and ultimately cell function. Furthermore, LINE-1 elements regulate replication and insertion of short interspersed nuclear elements such as Alu. Alu insertion into genes can also regulate gene expression and/or function (Wallace et al. 2008a, 2008b). Such findings suggest that these retrotransposons may represent yet another level of epigenetic regulation of gene expression. Indeed, a growing body of evidence suggests that LINE-1 transposition plays a critical role both in cell differentiation and in regulating the mammalian transcriptome (Coufal et al. 2009; Zemojtel et al. 2007). Changes in methylation of LINE-1 secondary to environment exposures may have significant impacts on health (Wallace et al. 2008a, 2008b; Wilson et al. 2007). Within this context, our finding that bone lead levels are associated with lower LINE-1 element DNA methylation may have direct, rather than indirect, implications for the mechanistic role of lead exposure on health during aging. Future work targeting methylation at specific, intact LINE-1 sequence is needed to test this possibility.

### Use of DNA methylation changes in WBCs as a biomarker

The growing interest in epigenetic biomarkers is due to their potential to explain fetal origins of disease or, even more broadly, to explain the latency between exposure to toxic substances and subsequent disease phenotypes. Measures of DNA methylation hold substantial promise as a biomarker for toxic environmental exposures, but because such marks are specific to cell type, much work is still needed to determine what role, if any, WBC DNA methylation may have for diseases that occur in inaccessible target tissues, such as the brain. Measuring target organ DNA methylation would be ideal, but in many human studies obtaining such measures is not feasible. Nevertheless, peripheral WBC DNA methylation might hold promise as an exposure biomarker (as opposed to a biomarker of effect) by demonstrating that environmental factors can alter WBC DNA methylation patterns. LINE-1 and Alu are relatively primitive markers of global methylation because they are averaged across thousands of repetitive sequences. A more specific epigenomic measure, such as patterns of DNA methylation within gene-specific promoter sequences, might yield a more specific pattern quantifying past lead exposure. This finding raises the question of whether DNA methylation patterns could be a methodology to reconstruct past chemical exposure.

Recently, Chanda et al. (2006) observed significant hypermethylation of the p16 gene promoter in DNA from peripheral blood lymphocytes of subjects exposed to arsenic-contaminated water, showing that DNA from peripheral blood cells may be a suitable tissue for the development of epigenetic biomarkers. An expansion of this concept (i.e., that gene-specific methylation is altered by environment) to epigenomics may allow investigators to determine whether signature patterns of DNA methylation occur across the WBC epigenome that are specific to exposure to certain types of chemicals. This is effectively the concept underlying the field of proteomics. Epigenomics, because its marks are stable but modifiable and because the technology is already highly developed, may be an alternative means for such biomarker development. Because WBCs are not a target for many diseases, changes in DNA methylation from peripheral blood cells may not reflect mechanistic pathways in the target tissue. However, our results suggest that WBC DNA methylation may be useful as a biomarker of exposure, possibly allowing investigators to confirm whether exposures are chronic versus short term. Given that exposure assessment is the “Achilles heel” of environmental epidemiology, particularly with respect to case–control studies of chronic disease, the potential for epigenetic or epigenomic marks to establish or confirm the presence or chronicity of environmental exposures deserves further study.

### Limitations and future research

Our understanding of the role of DNA methylation and other epigenetic marks on gene expression and disease is clearly rudimentary at this stage. It is difficult therefore to ascribe the direct consequences of our findings to specific lead-associated health effects. We believe that epigenetic marks may play a significant role in the biological pathway from environmental exposures to disease and health states, and perhaps are critical to explaining the long latencies often observed between lead exposure and health effects. However, we also believe that to fully interpret such data, we must first establish norms and whenever possible study the target organ directly, rather than WBCs. Unlike DNA sequence, for which a large inventory of data has established a consensus baseline sequence, there is no blueprint of what are “normal” DNA methylation patterns. Such an inventory will be complex, as it is cell type–specific and will also change with different life stages. Establishing baseline patterns of methylation across the epigenome in tissues readily accessible to researchers (e.g., blood, saliva, buccal cells) will be critical to understanding the relationship between environmental exposures, DNA methylation changes, and health. Because methylation patterns are not inert, inventories will need to account for changes in methylation patterns specific to life stages. Work conducted concurrently in animal models may also allow us to better understand how changes in WBC DNA methylation in response to environmental stimuli correlate to changes in target tissues/cells that are not readily accessible, such as brain regions. In summary, we have found that LINE-1 DNA methylation is inversely associated with patella lead levels in a population of elderly men. Our work suggests that changes in DNA methylation may be part of the underlying biological pathway between lead exposure and multiple lead-associated health effects. There are many caveats to our findings, including whether findings in WBC DNA are representative of target tissue changes and whether the association of lead with decreased LINE-1 methylation is functional or merely representative of the impact of lead on DNA methylation globally. Despite these limitations, we believe that a better understanding of epigenetic marks as they relate to environmental exposure is critical to our understanding of human health and development. Future work is needed to establish baseline norms for epigenetic marks, to establish whether specific environmental exposures alter such marks, to determine whether WBC DNA methylation changes correlate with target tissue changes, and finally, to determine whether such changes are part of the causal pathway between environment and disease.

## Figures and Tables

**Table 1:** Summary statistics [mean ± SD or *n* (%)] at baseline for participants with and without bone lead measurements in the NAS.

Characteristic	With bone lead measure (*n* = 517)	Without bone lead measure (*n* = 162)	*p-Value*^a^
Tibia lead (g/g)	20.5 ± 14.8	—	
Patella lead (g/g)	27.4 ± 19.7^b^	—	
Blood lead (μg/dL)	4.1 ± 2.4^c^	3.9 ± 2.1^d^	0.59
Alu (%)	26.3 ± 1.0	26.2 ± 1.2	0.27
LINE-1 (%)	76.8 ± 1.9	77.1 ± 2.1	0.16
Age (years)	72.4 ± 6.5	72.5 ± 7.6	0.95
BMI (kg/m^2^)	28.0 ± 3.8	28.5 ± 4.5	0.45
Lymphocytes (%)	25.5 ± 8.6	25.7 ± 7.2	0.56
Pack-years of cigarettes	20.1 ± 25.3	25.6 ± 32.7	0.02
Education			0.85
Less than high school diploma	46 (8.9)	10 (6.2)	
High school diploma/some college	314 (60.7)	88 (54.3)	
Four years of college	143 (27.7)	39 (24.1)	
Missing	14 (2.7)	25 (15.4)	

a *p*-Values for differences between subjects with and without bone lead measurements from Wilcoxon rank sum or Fisher’s exact test.

b *n* = 508 for patella and 517 for tibia.

c *n* = 463.

d *n* = 127.

**Table 2:** Distribution of DNA methylation at baseline by categories of covariates.

		Methylation (mean ± SD)	
Variable	*n* (%)	Alu (%)	LINE-1 (%)
Tibia lead (g/g)^a^			
13.0	166 (32.1)	26.40 ± 0.95	77.01 ± 1.79
> 13.0 to 22	176 (34.0)	26.18 ± 1.02	76.64 ± 2.02
> 22	175 (33.9)	26.32 ±1.04	76.73 ± 1.74
*p* for trend		0.50	0.18

Patella lead (g/g)^a^			
17.0	167 (32.9)	26.36 ± 1.03	77.17 ± 1.79
> 17.0 to 29	160 (31.5)	26.30 ± 0.91	76.61 ± 1.94
> 29	181 (35.6)	26.22 ± 1.07	76.57 ± 1.80
*p* for trend		0.19	0.003

Blood lead (g/dL)^a^			
2.0	114 (24.6)	26.35 ± 0.85	76.97 ± 1.78
> 2.0 to 4.0	205 (44.3)	26.18 ± 0.95	76.68 ± 2.06
> 4.0	144 (31.1)	26.41 ± 1.16	76.78 ± 1.61
*p* for trend		0.52	0.44

Lymphocytes (%)^a^			
21.0	165 (31.9)	26.26 ± 0.95	77.03 ± 1.88
> 21.0 to 28	186 (36.0)	26.39 ± 1.08	76.69 ± 1.64
> 28	166 (32.1)	26.23 ± 0.98	76.66 ± 2.05
*p* for trend		0.79	0.07

Age (years)			
< 70	186 (36.0)	26.41 ± 0.97	77.05 ± 2.01
70–79	265 (51.3)	26.28 ± 1.03	76.64 ± 1.80
> 80	66 (12.8)	26.07 ± 1.01	76.65 ± 1.54
*p* for trend		0.02	0.04

BMI (kg/m^2^)			
< 25	97 (18.8)	26.22 ± 0.98	76.74 ± 1.89
25–30	290 (56.1)	26.28 ± 0.94	76.72 ± 1.85
> 30	130 (25.1)	26.39 ± 1.16	76.97 ± 1.85
*p* for trend		0.21	0.31

Education			
Less than high school	46 (9.2)	26.28 ± 1.05	76.07 ± 1.79
High school/some college	314 (62.4)	26.25 ± 0.97	76.85 ± 1.84
Four years of college	143 (28.4)	26.40 ± 1.09	76.88 ± 1.88
*p* for trend		0.25	0.06

Cigarette smoking (pack-years)			
0	174 (33.7)	26.22 ± 0.99	76.67 ± 1.58
30	206 (39.8)	26.40 ± 1.09	76.71 ± 1.99
> 30	137 (26.5)	26.25 ± 0.89	77.05 ± 1.96
*p* for trend		0.68	0.09

a Categories are based on the tertile of the variable.

**Table 3:** Cross-sectional lead biomarker association with DNA methylation estimated from mixed-effects models with increasing adjustment for confounders.

	Alu β (95% CI)	LINE-1 β (95% CI)
Tibia lead (IQR = 15 g/g)		
Model 1 (*n* = 787)	0.01 (−0.08 to 0.10)	−0.11 (−0.28 to 0.05)
Model 2 (*n* = 762)	0.01 (−0.09 to 0.11)	−0.06 (−0.23 to 0.12)
Model 3 (*n* = 694)	0.02 (−0.10 to 0.13)	−0.07 (−0.29 to 0.14)

Patella lead (IQR = 19 g/g)		
Model 1 (*n* = 772)	−0.01 (−0.10 to 0.07)	−0.20 (−0.36 to −0.05)*
Model 2 (*n* = 747)	−0.01 (−0.10 to 0.08)	−0.17 (−0.33 to 0.00)*
Model 3 (*n* = 679)	−0.03 (−0.14 to 0.08)	−0.25 (−0.44 to −0.05)*

Blood lead (IQR = 2 g/dL)		
Model 1 (*n* = 716)	0.03 (−0.04 to 0.11)	−0.01 (−0.15 to 0.13)
Model 2 (*n* = 694)	0.03 (−0.05 to 0.10)	0.04 (−0.10 to 0.19)

Abbreviations: CI, confidence interval; IQR, interquartile range. Model 1 was adjusted for age, BMI, and percent lymphocytes; model 2 was adjusted for age, BMI, percent lymphocytes, education, and pack-years of cigarettes; and model 3 was adjusted for for age, BMI, percent lymphocytes, education, pack-years of cigarettes, and blood lead levels.

* *p* ≤ 0.05; *n* for each model represents total observations rather than individuals.
